# Mental health, self-rated health, risky sexual behaviour, alcohol use, and drug use among students who intend to spend a semester abroad – a cross-sectional study

**DOI:** 10.3389/fpubh.2023.1116497

**Published:** 2023-06-29

**Authors:** Emil Danehorn, Ulla Peterson, Marie Oscarsson, Goldina Smirthwaite, Katarina Swahnberg

**Affiliations:** ^1^Faculty of Health and Life Sciences, Linnaeus University, Kalmar, Sweden; ^2^Faculty of Arts and Social Sciences, Karlstad University, Karlstad, Sweden

**Keywords:** mental health, sexually risky behaviour, students, alcohol use, drug use, health

## Abstract

**Aim:**

Our aim was to investigate potential differences in mental health, self-rated health, risky sexual behaviour, alcohol use, and drug use between (1) Prospective exchange students and campus students separated by sex, and (2) male and female students as a group.

**Method:**

Comparative cross-sectional design using an online survey containing the following instruments: Knowledge, Attitudes, and Sexual Behaviour in Young People in Sweden; Self-Rated Health Questionnaire; and General Health Questionnaire 12. One-hundred and fourteen prospective exchange students and 451 campus students participated in the study.

**Results:**

Male prospective exchange students rated their mental health as being better and had used cannabis more often compared with female prospective exchange students. Male prospective exchange students also rated their mental health as being better than male campus students. Female students, in general, rated their mental health as worse than male students. A larger proportion of male prospective exchange students had sex together with alcohol compared with male campus students, and a larger proportion of female prospective exchange students had sex with a new partner and drank more alcohol compared to female campus students.

**Conclusion:**

The result shows that risky alcohol use and sexually risky behaviour is prominent amongst prospective exchange students. It is possible that they will continue, and even increase their risky behaviour whilst abroad as they find themselves in a new social context, and free from influence of the rules and restrictions that they might have at home. With limited knowledge of the local culture, native language, and in an unfamiliar environment, it is possible that the risks will be enhanced and possibly decrease their health. This highlights the need for proactive interventions, conceivably with some variations in content between sexes.

## Introduction

Internationalisation is a vital activity for universities these days and it is often used as a tool to increase the quality of the education, university status, and academic recognition, and exchange studies and mobility programmes play a natural and important role in this process (1). For university students, this means the opportunity to spend a semester abroad as exchange students. Approximately 24,000 Swedish students from different universities spent a semester abroad, and approximately 38,000 exchange students from different countries travelled to Sweden in the 2018/2019 school year (2).

Even though many Swedish students choose to spend a semester abroad (hereafter referred to as Swedish exchange students), there has been limited research on this group. The few studies that have been carried out, have focused on risky behaviour, which seems to be prominent amongst Swedish exchange students (3, 4).

It has been shown that 36% of Swedish exchange students consume alcohol in a way that could be associated with increased risks for ill-health (3). This is higher than the general Swedish population between the ages of 16 and 29, where approximately 21% have a risky consumption of alcohol (5). However, lower then Swedish university students in general, where 46.1% of male and 44% of female students between the ages of 18 and 24 consume alcohol at a risky level (6). In addition, 43% of male and 23% of female Swedish university students between the years 21 to 25 years binge drink alcohol at least once per week (7).

Even though it is common to quantify alcohol into risky and non-risky consumption for research purposes, the use of alcohol is always associated with an increased risk for ill-health and there is no such thing as risk-free alcohol consumption. However, four or more glasses at a single occasion or 10 glasses per week is associated with significantly increased risks for ill-health, not only the development of addiction, but also accidents and diseases (8).

Swedish exchange students’ risky alcohol consumption is also associated with having sex with a new partner; 20% met a new partner whilst abroad but only 65% used a condom at every sexual encounter (3). It has also been shown that 51% of Swedish exchange students have had sex with a new partner during their exchange studies, and 87% of them had engaged in risky sexual behaviour, such as sex together with alcohol and sex without protection (4).

A risky sexual behaviour can have several components, but common definitions are sex that increases the risk of sexually transmitted infection (STI), such as sex with multiple partners, sex without protection, and/or sex together with drugs and alcohol (9). The use of protection seems to be higher for exchange students (3), than for the Swedish population in general, where 50% of women and 46% of men between the ages of 20 and 24 years did not use a condom the last time they had sex with a new, unknown partner. Common reasons for not using condoms were alcohol use, no condoms available, that they reduce sensitivity, and unplanned sexual activities (9–11).

Swedish exchange students rate their mental health as good (4), however, research on non-Swedish exchange students shows that exchange studies might be an overwhelming experience because the students might not be fully prepared for the journey, and this can have a negative effect on their mental health (12). A number of symptoms were identified in Asian exchange students, including sleeping problems, depressive symptoms (13), anxiety, stress (14), and psychological distress (15). Common causes for decreased mental health in Asian exchange students were lack of social contacts, homesickness (16), academic stress, and social isolation (17). Examples of decreased mental health have also been shown amongst exchange students from the US, where 44% experienced reduced well-being due to stress or emotion-related problems (18). Both a high alcohol consumption and mental ill-health can also lead to lower levels of academic functioning amongst exchange students (19, 20).

Studies on Swedish exchange students suggest that they indulge in sexually risky behaviour and use alcohol in a risky way, which might lead to an increased risk for accidents and diseases (8, 9). In addition, studies on non-Swedish exchange students suggest that there is a risk for mental ill-health whilst abroad (12–18, 20). These risk factors could possibly be enhanced due to exchange students being in an unknown environment, with limited knowledge of the native language and healthcare access. In order to adjust measures to better fit the majority of the Swedish exchange students and design proactive measures to ensure academic performance, good health, and decreased risky sexual behaviour and alcohol consumption, it could be fruitful to investigate whether, and if so how, students who intend to spend a semester abroad differ from students who remain on campus. Students who intend to spend a semester abroad are hereafter referred to as prospective exchange students. Students in the comparison group are hereafter referred to as campus students.

### Aim

Our aim was to investigate potential differences in mental health, self-rated health, risky sexual behaviour, and alcohol and drug use between:

1. Prospective exchange students and campus students separated by sex.
2. Male and female students as a group.

## Materials and methods

### Sample and procedure

Participants were all prospective exchange students at a Swedish university in 2018 who were going to spend a semester abroad within 3 months, and a group of campus students in semesters four and five who formed the comparison group. The ratio for the comparison group was set at one to five, a ratio of more than one to four has limited effect on power (21). However, we were worried that the response rate would be low in the comparison group, and therefore concluded that one to five would be a suitable compromise. In May 2018, a web-survey was sent out to 200 prospective exchange students and 1,000 campus students, and in November 2018 to 183 prospective exchange students and 915 campus students.

The study design was a cross-sectional comparative study based on a web survey that was sent out in 2018 to 2,298 students at a Swedish university. Along with the survey, a letter was attached with information about the aim of the study, that participation was anonymous and voluntary, and how the data were going to be handled. Two reminders were sent within a two-week interval. The student department provided the email addresses for the prospective exchange students and the campus students.

### Measures

The web survey contained a single item from the Short Form Health Survey (22), the General Health Questionnaire 12 (GHQ12) (23), and the Knowledge, Attitudes and Sexual Behaviour in Young People in Sweden survey (UngKAB) (24).

The UngKAB survey was used to investigate sexual behaviour, drug use, and alcohol use (24). UngKAB is an online survey originally initiated by the social health department in Sweden, targeting young males and females aged 15–29 years. In this survey, 12 items about sexual behaviour and alcohol and drug use were used. The questions about drugs and alcohol could be answered as follows: daily, a few times a week, a few times a month, more rarely, and never. Questions about sexual behaviour could be answered as yes/no or, in some cases, with numerals, for example, the number of sexual partners during the last six months. Questions about sexual behaviour included yes/no questions about sex without protection against STI, sex together with alcohol, and sex together with alcohol and without protection against STI.

Self-rate health was measured with a single item from the Short Form Health Survey (22). The instrument has previously been used in the general Swedish population with participants between 25 and 34 years with satisfactory results (25); it has also been used by the World Health Organisation (WHO) (26). In this study, participants were asked to rate their health as excellent, very good, good, fair, or poor (27). There are variations in self-rated health; for instance, the WHO uses a slightly different wording, where “fair” is the third option and is considered neutral (26), and other studies have interpreted fair, poor, and very poor as poor health (28). In this study, responses of excellent, very good, and good were considered good self-rated health, as they were positively worded, whilst responses of fair and poor were considered poor self-rated health.

The GHQ12 (23) was used to measure mental health and has been validated in Swedish contexts (29). It was originally developed to identify mental conditions such as stress, anxiety, and depression, with the exception of chronic psychiatric diseases. The GHQ12 consists of 12 questions regarding Being able to concentrate, Losing sleep over worry, Playing a useful role in things, Being capable of making decisions, Being constantly under strain, Not being able to overcome difficulties, Enjoying day-to-day activities, Facing one’s problems, Feeling unhappy and depressed, Lacking self-confidence, Thinking of oneself as worthless, and Being reasonably happy. The response alternatives of “Better than usual,” “Same as usual,” “Worse than usual,” and “Much worse than usual” were given scores of 0–3, respectively. Six questions are negatively worded. In this study, the GHQ12 was treated as a continuous scale and was not given any cut-off points; however, lower scores indicate better mental health. Cronbach’s alpha was 0.85 for male students and 0.84 for female students.

### Statistical analysis

Since UngKAB and self-rated health are binomial, we compared the proportions between the responses to find statistically significance differences between male and female as well as prospective exchange and campus students. Statistical significance was tested with a 95% confidence interval. To find statistical significance between the means in GHQ12, we used non-parametric statistics due to the small sample size, independent groups and the Likert scale coding of the responses in GHQ12. The Mann–Whitney U-test was performed with a value of *p* of 0.05.

### Ethical considerations

The study and all methods were approved by the Regional Ethical Review Board, Linköping (Dnr 2017/504–3). All methods were carried out in accordance with relevant guidelines and regulations, and the ethical principles of the Declaration of Helsinki. Detailed information about the content of the survey as well as the aim of the study were included in the information letter and survey instructions and respondents were guaranteed confidentiality. We therefore assumed that those who, for any reason, did not want to participate in the survey, abstained from participating. Completing and submitting the online questionnaire was considered informed consent according to the approved protocol.

## Results

The response rate for the survey that was sent out in May was 24.5% (*n* = 49) for the prospective exchange students and 23.1% (*n* = 231) for the campus students. In the survey that was sent out in November the response rate was 36.1% (*n* = 66) for the prospective exchange students and 24.5% (*n* = 224) for the campus students. Five students who had responded as gender other than male or female were excluded because there were too few of them to form a separate group. The mean ages of the male prospective exchange students, female prospective exchange students, male campus students, and female campus students were 25, 24.3, 26.4, and 28 years, respectively.

### Mental health

There was a significant difference in mental health between male prospective exchange students (GHQ12 = 9.7) and male campus students (GHQ12 = 12.6) (*p* = 0.003), but there was no significant difference between female prospective exchange students (GHQ12 = 12.4) and female campus students (GHQ12 = 13.5) (*p* = 0.11). The analysis showed a statistically significant difference in mental health between male students (GHQ12 = 11.7) and female students (GHQ12 = 13.3) (*p* = 0.01), where female students rated their mental health as worse than male students. There was also a statistically significant difference between male prospective exchange students (GHQ12 = 9.7) and female prospective exchange students (GHQ12 = 12.4) (*p* = 0.03).

### Self-rated health

Male prospective exchange students rated their health as excellent, very good, or good (94.1%) to a higher degree than male campus students (81.6%), ∆ (95% CI) 12.6 (0.7, 22), whilst there were no significant differences between female prospective exchange (90.5%) and female campus students (80.7%), ∆ (95% CI) 9.7(−0.7; 16.5). There were no statistically significant differences in self-rated health between male and female prospective exchange students or between male and female campus students. In addition, there were no statistically significant differences in self-rated health between male students and female students as a group (Table 1).

**Table 1:** Self-rated health divided into sex, prospective exchange students, and campus students (*N* = 565).

	Prospective exchange students		Campus students		Prospective exchange students		Campus students	
	Male	%	Male	%	Female	%	Female	%
	*n* = 51		*n* = 103		*n* = 63		*n* = 348	
Self-rated health								
Excellent	9	17.6	10	9.7	5	7.9	26	7.5
Very good	23	45.1	35	34	29	46	130	37.4
Good	16	31.4	39	37.9	23	36.5	125	35.9
Fair	3	5.9	19	18.4	5	7.9	53	15.2
Poor					1	1.6	14	4

### Drugs and alcohol

Female prospective exchange students consumed more alcohol on the same occasion than female campus students, although not more frequently. There were no significant differences between male students (Table 2). More male prospective exchange students (29.4%) had used cannabis compared with female prospective exchange students (7.9%) ∆ (95% CI) 21.5 (7.3; 35.8). There were no statistically significant differences between the sexes in terms of how often the students had consumed alcohol over the last six months. A higher number of male students (18.8%) had used cannabis compared to the female students (6.1%), ∆ (95% CI) 12.8 (6.7; 19.9), and male students (6.5%) had used other substances such as amphetamines, GHB, cocaine, spice, or other drugs to a higher degree compared to female students (2.4%) in total, ∆ (95% CI) 4.1 (0.5; 9.2).

**Table 2:** Comparison of the use of alcohol and other drugs divided into sex, prospective exchange students, and campus students (*N* = 565).

	Prospective exchange students		Campus students			Prospective exchange students		Campus students		
	Male	%	Male	%	▹%	Female	%	Female	%	▹%
	*n* = 51		*n* = 103			*n* = 63		*n* = 348		
Use of cannabis in past 6 months										
Daily	1	2.0	2	1.9	0.05 (−5.1; 8.5)		0.0	1	0.3	0.3 (−5.5; 1,6)
A few times a week	2	3.9		0.0	3.9 (−0.7; 13.2)	1	1.6	1	0.3	1.3 (−0.6; 8,2)
A few times a month		0.0	2	1.9	1.9 (−5.2; 6.8)		0.0	3	0.9	0.9 (−4.9; 2,5)
More rarely	12	23.5	12	11.7	11.9 (−0.3; 26)	4	6.3	15	4.3	2 (−2.7; 11,1)
Never	**36**	**70.6**	**87**	**84.5**	**13.9 (0.4; 28.6)**	58	92.1	328	94.3	2.2 (−3.2; 11,7)
Use of other drugs in past 6 months										
Daily								1	0.3	0.3 (−5.5; 1,6)
A few times a week										
A few times a month	2	3.9			3.9 (−0.7; 13.2)	1	1.6			1.6 (−0.1; 8,5)
More rarely	5	9.8	3	2.9	6.9 (−0.8; 18.2)		0.0	8	2.3	2.3 (−3.6; 4,5)
Never	**44**	**86.3**	**100**	**97.1**	**10.8 (2.1; 23)**	62	98.4	339	97.4	0.1 (−6; 3.6)
Alcohol consumption in the past 6 months										
Daily										
A few times a week	11	21.6	27	26.2	4.6 (−10.4; 17.6)	14	22.2	79	22.7	0.5 (−11.9; 10.2)
A few times a month	16	31.4	38	36.9	5.6 (−10,6; 20.2)	28	44.4	129	37.1	7.4 (−5.3; 20.6)
More rarely	20	39.2	27	26.2	13 (−2.3; 28.6)	17	27.0	99	28.4	1.5 (−11.4; 12.1)
Never	4	7.8	11	10.7	2.8 (−8.8; 11.7)	4	6.3	41	11.8	5.4 (−3.9; 10.9)
Standard glasses of alcohol										
1 to 2	4	7.8	22	21.4	13.5 (−0.9; 23.6)	**4**	**6.3**	**110**	**31.6**	**25.3 (15.2; 31.6)**
3 to 4	7	13.7	20	19.4	5.7 (−7.9; 16.8)	**10**	**15.9**	**99**	**28.4**	**12.6 (0.8; 21.2)**
5 to 6	14	27.5	29	28.2	0.7 (−14.9; 14.6)	**27**	**42.9**	**69**	**19.8**	**23 (10.7; 35.9)**
7 to 9	12	23.5	13	12.6	10.9 (−1.4; 25.1)	**16**	**25.4**	**34**	**9.8**	**15.6 (5.8; 27.9)**
10 or more	7	13.7	10	9.7	4 (−6; 16.8)	**5**	**7.9**	**5**	**1.4**	**6.5 (1.6; 15.9)**
Does not drink alcohol	7	13.7	9	8.7	4.9 (−4.9; 17.7)	1	1.6	31	8.9	7.3 (−0.01; 11)

Bold text indicates a significant difference.

▹% indicates proportional difference.

### Risky sexual behaviour

More male prospective exchange students (73.3%) had sex together with alcohol compared with male campus students (54.8%). More female prospective exchange students (41.4%) had sex with a new partner during the last six months compared to female campus students (20.1%). Both male (25.5%) and female (19.1%) prospective exchange students had had sex with more than three partners during the last six months compared with male (9.7%) and female (4.6%) campus students (Table 3). There were no significant differences between the male and female prospective exchange students regarding sex with a new partner, sex together with alcohol, or the use of protection against STI.

**Table 3:** Comparison of sexual behaviours divided into sex, prospective exchange students, and campus students (*N* = 565).

	Prospective exchange students		Campus students			Prospective exchange students		Campus students		
	Male	%	Male	%	▹%	Female	%	Female	%	▹%
	*n* = 51		*n* = 103			*n* = 63		*n* = 348		
Number of sexual partners in the past 6 months										
None	11	21.6	35	34	12.4 (−3.1; 25.6)	5	7.9	60	17.2	9.3 (−0.01; 15.5)
1 to 3	27	56.3	58	52.9	3.4 (−12.8; 19.6)	46	73	272	78.2	5.2 (−5.3; 17.8)
>3	**13**	**25.5**	**10**	**9.7**	**15.8 (3.5; 29.9)**	**12**	**19.1**	**16**	**4.6**	**14.5 (6.2; 25.9)**
Sex with new partner in the past 6 months										
Yes	22	43.1	30	35.7	13.2 (−4.4; 30.1)	**24**	**41.4**	**66**	**20.1**	**21.3 (8.6; 34.7)**
No	23	45.1	54	64.3		**34**	**58.6**	**262**	**79.9**	
STI protection, sex with a new partner										
Yes	11	50.0	14	46.7	3.3 (−22.5; 28.7)	13	54.2	30	45.5	8.7 (−13.8; 30)
No	11	50.0	16	53.3		11	45.8	36	54.5	
Alcohol together with sex in the past 6 months										
Yes	**33**	**73.3**	**46**	**54.8**	**18.6 (0.1; 33.7)**	47	81.0	195	59.5	4.5 (−5.1; 17)
No	**12**	**26.7**	**38**	**45.2**		11	19.0	133	40.5	
STI protection, alcohol together with sex										
Yes	12	36.4	10	21.7	14.6 (−5.2; 34.1)	17	36.2	58	29.7	6.4 (−7.5; 21.9)
No	21	63.6	36	78.3		30	63.8	137	70.3	

Bold text indicates a significant difference.

▹% indicates proportional difference.

## Discussion

Male prospective exchange students rated their mental health as being better and had used cannabis more often compared with female prospective exchange students. More female prospective exchange students had sex with a new partner during the last six months and drank higher levels alcohol on the same occasion compared to female campus students. Male prospective exchange students had had more sex together with alcohol, and rated their mental health and health as better than male campus students. Female students rated their mental health as worse than male students. A higher number of male prospective exchange students had used cannabis, amphetamines, GHB, cocaine, spice, or other drugs than male campus students.

There were no significant statistical differences in alcohol consumption between the groups, with the exception that female prospective exchange students drank more alcohol per occasion than female campus students; however, the overall use of alcohol was high in comparison with the general Swedish population between the ages of 16 and 29 (5), but similar to exchange students in the US where 31% of the exchange students drank alcohol weekly (30). Previous research has also shown that at least one-third of Swedish exchange students drink alcohol in a way that could be associated with increased risks for ill-health (3). Moreover, Swedish university students in general consume alcohol at a high level (31–34), as well as Norwegian students (35, 36). Binge drinking is also common for Swedish university students in general (7).

Exchange students in Europe and Australia reported that both general use of alcohol and binge drinking increased to a level higher than local students whilst abroad (37, 38). It was also shown that exchange students may adapt to the country’s local drinking culture in order to be included in social life (37). Therefore, it is possible that Swedish students will also increase their alcohol consumption, from an already high level. Moreover, frequent drinking and binge drinking have been shown to increase the risk for anxiety and depression amongst students in general (36, 39).

However, previous research on exchange students show that they experience both good mental health and high self-esteem (4). This could indicate that when the prospective exchange students go on their exchange trip, generally, their mental health is not negatively affected. However, there are still risk factors that could potentially lead to a decrease in mental health (12, 13, 15, 17), which should be considered when preparing to go on an exchange trip.

The female students in our study rated their mental health as worse than male students. This is in line with current statistics from the general Swedish population between 16 and 29 years, where it was shown that 20% of women and 10% of men experienced severe worry and/or anxiety (40). There is an increasing demand from society that women should be successful in school and work, which contributes to stress (25, 41). Swedish women also use social media more frequently than men and are exposed to cyber bullying to a higher degree (41). Women between 18 and 34 years who regularly use social media report significantly lower mental health than women who do not use or rarely use social media (42). It is possible that the use of social media could increase further whilst abroad, in attempt to stay in contact with family, friends and cope with loneliness. These additional risk factors (25, 41, 42) may indicate that female students are at a higher risk of worsening mental ill-health whilst abroad.

Although female students did not report worse self-rated health than male students, there are factors that might negatively affect self-rated health whilst abroad. Previous research has shown that academic pressure and being behind in school work has a negative effect on self-rated health (43). Self-rated health also has a general tendency to reduce after starting at a university, with increased stress as a contributing factor (44). The unavailability of healthcare services has also been shown to have a negative effect on students self-rated health (45). This, combined with academic pressure (43–45), could possibly be associated with a semester in a foreign country, and lead to reduced self-rated health amongst exchange students.

Our results showed that a higher proportion of the female prospective exchange students had had sex with a new partner at least once over the last six months; it was also shown that only about half of them used a condom whilst having sex with a new partner. Moreover, previous research on Swedish exchange students showed one-third did not use a condom whilst having sex with a new partner whilst abroad (4). The frequency of prospective exchange students condom-use is higher than in the general Swedish population between the ages of 20 and 24 (9). However, it is similar to other students in Greece (46), but differs somewhat from students in China, where only one-quarter always use a condom (47). Students in South Africa, however, did use condoms whilst having sex with a new partner more often than Swedish prospective exchange students (48).

A common reason for not using condoms whilst having sex is the perception that condoms reduce sensitivity (9–11). Other factors are unplanned sexual activity (10), no condoms available (9), and embarrassment at obtaining condoms (49). Alcohol also seems to be a factor in reducing willingness to use condoms. Research suggests that heavy use of alcohol is strongly associated with decreased intentions of using a condom whilst having sex (50), and there is a link between alcohol use and casual sexual encounters amongst university students (51). Research on tourists suggests that sex together with alcohol or other drugs whilst abroad decreases the likelihood of using protection (52). In our study, the use of condoms whilst having sex together with alcohol was low. This is similar to a university in Greece, where students who used alcohol also showed a lower tendency to use a condom whilst having casual sex (46). Research also suggests that whilst abroad, Swedish exchange students indulge in sexually risky behaviour, such as sex together with alcohol and sex without using a condom (3, 4).

The high degree of sexually risky behaviour, such as more sexual partners and sex together with alcohol, may be due to Swedish exchange students having high self-esteem (4). A high level of self-esteem can bolster the desire to experiment with sex, alcohol, and drugs, which could lead to risky sexual behaviour (53). It has also been shown that low-self-esteem, anxiety and phobic anxiety is associated with a lower intention to use a condom amongst students (11, 54). The results also showed that male prospective exchange students had used cannabis more frequently, and previous research has shown that students who use cannabis have sex without protection more frequently than students who abstain from cannabis (55). There is also a social expectation for university students to drink alcohol and indulge in risky sexual behaviour (56).

For many students, exchange studies might be a totally new experience that can be challenging but also an opportunity for personal growth. However, exchange studies require commitment and a willingness to take risks, which may discourage many students (57). This could suggest that prospective exchange students are a select group of individuals who are resourceful in many ways but who are also not afraid to take risks. It is, however, possible that the exchange students’ risky behaviour could lead to more negative consequences whilst abroad compared to being at home, due to an unknown environment, new culture and social contacts.

As our result showed that sexually risky behaviour and high alcohol consumption were prominent amongst prospective exchange students, it could be beneficial to address these areas before departure. All Swedish universities have an established student health department, with a unique possibility to reach out to prospective exchange students to provide support and proactive measures to ensure good health. Examples of proactive measures from the student health department could be to; distribute free condoms, encourage students to look up locations for sexual health clinics in advance, and distribute preventive information about STI and safe sex prior to departure. Other examples could be to encourage prospective exchange students to investigate the local norms and cultural views on alcohol before departure and to locate healthcare facilities and police stations in advance. This includes learning the name of said facilities in the local language.

Generally, it can be difficult to prevent sexually risky behaviour by providing written information about safe sex, especially for exchange students (4). Further research is needed on how to reach out to prospective exchange students about safe sex and alcohol consumption.

### Methodological considerations

This study was carried out with instruments that have been used in studies with similar designs and populations (4), and we used a comparison group, which increased the credibility of our results (58). Cronbach’s alpha for the GHQ12 was above 0.8 for both male and female students, which also increases reliability. There was no missing data or internal loss, and all questions were satisfactorily answered. However, there are some limitations, and the results should be read with caution. A prominent weakness in this study was the overall low response rate, which could have affected the results. However, interest in answering surveys in general has decreased in recent years, which has led to an overall low response rate, especially in epidemiological self-reporting surveys (59). The low response rate could potentially have created a sampling bias, where the sample deviates from the population due to some individuals, with certain characteristics, being more likely to answer the survey. There is also a risk that the sample was not properly randomised thus creating a selection bias. Both sampling bias and selection bias might have caused the sample not to be representative for the population and thus might limit the generalisability of its results. Because this study included sensitive questions, it is also possible that the respondents answered the questions in a way that created a more positive image of themselves, perhaps a picture that reflected more strength or courage, and this might have caused social-desirability bias and thus skewed the results, which might also affect generalisation (60, 61). There has also been some critique against the single item from the Short Form Health Survey over the years, since it is a highly subjective instrument (62). However, the instrument has previously been shown to be a strong predictor for mortality (63, 64). It is also a widespread method and has previously been used on the general Swedish population with participants between 25 and 34 years with satisfactory results (25).

## Conclusion

The results show that risky alcohol use and sexually risky behaviour is prominent amongst prospective exchange students. It is possible that they will continue, and even increase, their risky behaviour whilst abroad as they find themselves in a new social context, and free from influence of the rules and restrictions they might have back home. With limited knowledge of the local culture, native language, and in an unfamiliar environment, it is possible that the risks will be enhanced and possibly decrease their health. This highlights the need for proactive interventions, conceivably with some variations in content between sexes.

## Data availability statement

The datasets presented in this study can be found in online repositories. The names of the repository/repositories and accession number(s) can be found at: https://snd.gu.se/sv/catalogue/study/2020-68.

## Ethics statement

The studies involving human participants were reviewed and approved by Regional Ethical Review Board, Linköping (Dnr 2017/504-3). Written informed consent was not provided because detailed information about the content of the survey as well as the aim of the study were included in the information letter and survey instructions. We therefore assumed that those who, for any reason, did not want to participate in the survey, abstained from participating. Completing and submitting the online questionnaire was considered informed consent according to the approved protocol by the Regional Ethical Review Board.

## Author contributions

All authors listed have made a substantial, direct, and intellectual contribution to the work and approved it for publication.

## Conflict of interest

The authors declare that the research was conducted in the absence of any commercial or financial relationships that could be construed as a potential conflict of interest.

## Publisher’s note

All claims expressed in this article are solely those of the authors and do not necessarily represent those of their affiliated organizations, or those of the publisher, the editors and the reviewers. Any product that may be evaluated in this article, or claim that may be made by its manufacturer, is not guaranteed or endorsed by the publisher.

## References

[1] Government Office of Sweden. En strategisk agenda för internationalisering - Delbetänkande av Utredningen om ökad internationalisering av universitet och högskolor, Nordsteds Juridik. (2018).

[2] Statistics Sweden. Higher education. International mobility in higher education from a Swedish perspective 2018/19. Stockholm: Swedish Higher Education Authority. (2019).

[3] Angelin M Evengård B Palmgren H. Illness and risk behavior in health care students studying abroad. Med Educ. (2015) 49:684–91. doi: 10.1111/medu.12753 26077216

[4] Petersson C Peterson U Swahnberg K Oscarsson M. Health and sexual behaviour amongst exchange students. Scand J Public Health. (2016) 44:671–7. doi: 10.1177/1403494816665753 27566998

[5] Public Health Agency of Sweden. Folkhälsans utveckling Årsrapport 2021. Östersund (2021).

[6] Public health agency of Sweden. Studenter och hög alkoholkonsumtion - Implementeringen av alkoholscreening och kort rÅdgivning pÅ studenthälsomottagningar. Stockholm (2014).

[7] Andersson A Wirén A Olvander C Ekman D Bendtsen P. Alcohol use amongst university students in Sweden measured by an electronic screening instrument. BMC Public Health. (2009) 9:229. doi: 10.1186/1471-2458-9-229, PMID: 19594906 PMC2724514

[8] Allebeck P Andreasson S Wålin S Ramstedt M Gripenberg J Damström-Takker K. Alkoholkonsumtion och risknivåer. Kunskapsunderlag och förslag till rekommendationer. Report 2018:1. Center for Epidemiology and Community medicin. (2018).

[9] Tikkanen M Abelsson J Forsberg M. UngKAB09 - a survey on knowledge, attitudes and behaviour amongst young people 16–29 years old. NW: Gothenburg university institution for social sciences (2011).

[10] Govindan B Maduravasagam K. Deploying machine learning to find out the reasons for not using condom in a questionnaire-based study of 120 patients. Indian J Sexually Transmit Diseases AIDS. (2018) 39:50–4. doi: 10.4103/ijstd.IJSTD_64_17, PMID: 30187027 PMC6111641

[11] Ruiz-Palomino E Gil-Llario MD Giménez-Garcia C Ballester-Arnal R. Explanatory psychological factors of inconsistently condom use amongst Spanish university students: gender differences. Span J Psychol. (2020) 23:23. doi: 10.1017/SJP.2020.14 32482177

[12] Forbes-Mewett H Sawyer AM. Mental health issues amongstst international students in Australia: perspectives from professionals at the coal-face In: Steven Threadgold EK, editor. Australian sociological association annual conference 2011. Newcastle: The Australian Sociological Association (TASA) (2011). 1–19.

[13] Kono K Eskandarich S Arai A Tamashiro H. Mental health and its associated variables among international students at a Japanese university: with special reference to their financial status. Immigrant Minority Health. (2014) 17:1654–9. doi: 10.1007/s10903-014-0100-1 25225076

[14] Cheung K Tam KY Tsang H Zhang LW Lit SW. Depression, anxiety and stress in different subgroups of first-year university students from 4-year cohort data. J Affect Disord. (2020) 274:305–14. doi: 10.1016/j.jad.2020.05.041, PMID: 32469820

[15] Lu SH Dear BF Johnston L Wootton BM Titov N. An internet survey of emotional health, treatment seeking and barriers to accessing mental health treatment amongst Chinese-speaking international students in Australia. Couns Psychol Q. (2014) 27:96–108. doi: 10.1080/09515070.2013.824408

[16] Lee J Koeske GF Sales E. Social support buffering of acculturative stress: a study of mental health symptoms amongst Korean international students. Int J Intercultioral Relations. (2004) 28:399–414. doi: 10.1016/j.ijintrel.2004.08.005

[17] Han X Han X Luo Q Jacobs S Jean-Babtiste M. Report of a mental health survey among Chinese international students at Yale University. J Am Coll Heal. (2012) 61:1–8. doi: 10.1080/07448481.2012.738267 23305539

[18] Hyun J Quinn B Madon T Lustig S. Mental health need, awareness, and use of counseling services among international graduate students. J Am Coll Heal. (2007) 56:109–18. doi: 10.3200/JACH.56.2.109-118 17967756

[19] Ansari WE Stock C Millis C. Is alcohol consumption associated with poor academic achievement in university students? Int J Prev Med. (2013) 4:1175–88. PMID: 24319558 PMC3843305

[20] Hunley HA. Students’ functioning whilst studying abroad: the impact of psychological distress and loneliness. Int J Intercult Relat. (2010) 34:386–92. doi: 10.1016/j.ijintrel.2009.08.005

[21] Woodward M. Study design and data analysis. 3rd ed. Sweden: Chapman Hall/CRC (2013).

[22] Sullivan M Karlsson J Ware JE. The Swedish SF-36 health survey. Evaluation of data quality, scaling assumptions, reliability and construct validity across general populations in Sweden. Soc Sci Med. (1995) 41:1349–58. doi: 10.1016/0277-9536(95)00125-Q, PMID: 8560302

[23] Goldberg D. The detection of psychiatric illness by questionnaire. A technique for the identification and assessment of non-psychotic psychiatric illness. London: Oxford University Press (1972).

[24] Tikkanen R Abelsson J Forsberg M. Study design –UngKAB09. Methodological considerations regarding the 2009 youth and sexuality survey in Sweden. Solna: The Swedish Institute for Communicable Disease Control (2011).

[25] Lidström MW Wennberg P Lundqvist R Forssén A Waller G. Time trends of comparative self-rated health in adults aged 25-34 in the northern Sweden MONICA study, 1990-2014. PLoS One. (2017) 12:e0187896. doi: 10.1371/journal.pone.0187896 29155858 PMC5695772

[26] Subramanian SV Huijts T Avendano M. Self-reported health assessments in the 2002 world health survey: how do they correlate with education? Bull World Health Organ. (2010) 88:131–8. doi: 10.2471/BLT.09.067058, PMID: 20428370 PMC2814481

[27] Public Health Agency of Sweden. National Public Health Survey. Stockholm (2016).

[28] Cislaghi B Cislaghi C. Self-rated health as a valid indicator for health-equity analyses: evidence from the Italian health interview survey. BMC Public Health. (2019) 19:533. doi: 10.1186/s12889-019-6839-5, PMID: 31072306 PMC6509759

[29] Lundin A Dalman C. Psykisk ohälsa mätt med the general health questionnaire. En validering i Stockholms län [mental illness measured with the general health questionnaire]. A validation in Stockholm County. (2020)

[30] Koyama C Belli G. Alcohol use, acculturative stress, and drinking motivation among international community college students. Multicult Counseling Dev. (2011) 39:229–40. doi: 10.1002/j.2161-1912.2011.tb00637.x

[31] Andersson C Johnsson KO Berglund M Öjehagen A. Alcohol involvement in Swedish university freshmen related to gender, age, serious relationship and family history of alcohol problems. Alcohol Alcohol. (2007) 42:448–55. doi: 10.1093/alcalc/agm008 17360719

[32] Nehlin C Öster C. Measuring drinking motives in undergraduates: an exploration of the drinking motives questionnaire revised in Swedish students. Subst Abuse Treat Prev Policy. (2019) 14:49. doi: 10.1186/s13011-019-0239-9, PMID: 31703747 PMC6839116

[33] Elgàn TH Durbeej N Gripenberg J. Breath alcohol concentration, hazardous drinking and preloading amongst Swedish university students. Nordic Stud Alcohol Drugs. (2019) 36:430–41. doi: 10.1177/1455072519863545, PMID: 32934577 PMC7434137

[34] Berman AH Andersson C Gajecki M Rosendahl I Sinadinovic K Bkankers M. Smartphone apps targeting hazardous drinking patterns amongst university students show differential subgroup effects over 20 weeks: results from a randomized. Controlled Trial Journclinical medicine. (2019) 8:1–20. doi: 10.3390/jcm8111807 PMC6912621 31661868

[35] Heradstvet O Skogen JC Brunborg GS Lønning KJ Sivertsen B. Alcohol-related problems amongst college and university students in Norway: extent of the problem. Scand J Public Health. (2021) 49:402–10. doi: 10.1177/1403494819863515, PMID: 31319770

[36] Erevik EK Pallesem S Torsheim T. Alcohol use amongst Norwegian students: demographics, personality and psychological health correlates of drinking patterns. Nordic Stud Alcohol Drugs. (2017) 34:415–29. doi: 10.1177/1455072517709918 PMC7450855 32934502

[37] Bhat P Dwyer R Vally H Pennay A. Acculturation and alcohol: exploring experiences of alcohol for Asian international students in Australia. Int J Intercult Relat. (2021) 85:184–90. doi: 10.1016/j.ijintrel.2021.09.011

[38] Dormal V Lannoy S Maurage P. Impact of exchange stay on alcohol consumption: longitudinal exploration in a large sample of European students. Alcohol Clin Exp Res. (2019) 43:1220–4. doi: 10.1111/acer.14028, PMID: 31034623

[39] Tembo C Burns S Kalembo F. The association between levels of alcohol consumption and mental health problems and academic performance amongst young university students. PLoS One. (2017) 12:e0178142. doi: 10.1371/journal.pone.0178142, PMID: 28658300 PMC5489147

[40] Public Health Agency of Sweden. Statistik psykisk hälsa: yngre vuxna 16–29 år. Stockholm (2022).

[41] Swedish Gender Equality Agency. Mental illness and other aspects of health 2021–2. Gothenburg (2021).

[42] Scott ES Canivet C Östergren P. Investigating the effect of social networking site use on mental health in an 18-34 year-old general population; a cross-sectional study using the 2016 Scania public health survey. BMC Public Health. (2020) 20:1753. doi: 10.1186/s12889-020-09732-z, PMID: 33225935 PMC7682097

[43] Porru F Schuring M Bültmann U Portoghese I Burdoff A Robroek SJW. Associations of university student life challenges with mental health and self-rated health: a longitudinal study with 6 months follow-up. J Affect Disord. (2022) 296:250–7. doi: 10.1016/j.jad.2021.09.057 34624809

[44] Martin SD Urban RW Johnson AH Magner D Wilson JE Zhang Y. Health-related behaviors, self-rated health, and predictors of stress and well-being in nursing students. J Prof Nurs. (2022) 38:45–53. doi: 10.1016/j.profnurs.2021.11.008, PMID: 35042589

[45] Macleod E Steenbeek A Bombay A. University students’ self-rated health and use of health services: a secondary analysis. Can J Nurs Res. (2019) 52:308–16. doi: 10.1177/0844562119870044 31412704

[46] Lori A Scott-Sheldon J Carey MP Carey KB. Alcohol and risky sexual behavior among heavy drinking college students. AIDS Behav. (2008) 14:845–53. doi: 10.1007/s10461-008-9426-9 18648928 PMC2982799

[47] Sun X Liu X Shi Y Wang Y Wang P Chang C. Determinants of risky sexual behavior and condom use amongst college students in China. AIDS Care. (2012) 25:775–83. doi: 10.1080/09540121.2012.748875, PMID: 23252705

[48] Mokgatle M Madiba S Cele L. A comparative analysis of risky sexual behaviors, self-reported sexually transmitted infections, knowledge of symptoms and partner notification practices amongst male and female university students in Pretoria, South Africa. Int J Environ Res Public Health. (2021) 18:15660. doi: 10.3390/ijerph18115660, PMID: 34070603 PMC8198344

[49] Oliveira AC Camarneiro AP Xavier BO Silva MA Simões IM Cardoso IM. Attitudes and embarrassment about condoms in nursing students. Acta paulista de enfermagem. (2020) 34:1–9. doi: 10.37689/acta-ape/2021AO01954

[50] Scott-Sheldon LA Carey MP Carey KB. Alcohol and risky sexual behavior among heavy drinking college students. AIDS Behav. (2008) 14:845–53. doi: 10.1007/s10461-008-9426-9 18648928 PMC2982799

[51] Cooper ML. Alcohol use and risky sexual behavior amongst college students and youth: evaluating the evidence. J Stud Alcohol. (2002) 14:101–17. doi: 10.15288/jsas.2002.s14.101 12022716

[52] Downing J Hughes K Bellis MA Calafat A Juan M Blay N. Factors associated with risky sexual behaviour: a comparison of British, Spanish and German holidaymakers to the Balearics. Eur J Pub Health. (2010) 21:275–81. doi: 10.1093/eurpub/ckq021 20231212

[53] Baumeister RF Campbell JD Kreuger JI Vohs KD. Does high self- esteem cause better performance, interpersonal success, happiness, or healthier lifestyles? Psychol Sci Public Interest. (2003) 4:1–44. doi: 10.1111/1529-1006.01431 26151640

[54] Yang N Xu Y Li S. Acculturative stress, poor mental health and condom-use intention amongst international students in China. Health Educ J. (2018) 77:142–55. doi: 10.1177/0017896917739443

[55] Buckner JD Lewis EM Shah SM Walukevich KA. Risky sexual behavior amongst cannabis users: the role of protective behavioral strategies. Addict Behav. (2018) 81:50–4. doi: 10.1016/j.addbeh.2018.01.039, PMID: 29425793

[56] Chanakira E O’Cathain A Goyder EC Freeman JV. Factors perceived to influence risky sexual behaviours amongst university students in the United Kingdom: a qualitative telephone interview study. BMC Public Health. (2014) 14:14. doi: 10.1186/1471-2458-14-1055 25300195 PMC4203964

[57] Bohman DM Borglin G. Student exchange for nursing students: does it raise cultural awareness’? A descriptive, qualitative study. Nurse Educ Pract. (2013) 14:259–64. doi: 10.1016/j.nepr.2013.11.006 24406034

[58] Bruchmann K. Compared to what? The importance of control groups in social comparison research. Basic Appl Soc Psychol. (2017) 39:91–100. doi: 10.1080/01973533.2017.1281808

[59] Galea S Tracy M. Participation rates in epidemiologic studies. Ann Epidemiol. (2007) 17:643–53. doi: 10.1016/j.annepidem.2007.03.013 17553702

[60] Jann B Höglinger M Diekmann A. Sensitive questions in online surveys: experimental results for the randomized response. Survey Res Methods. (2016) 10:171–87. doi: 10.1177/0049124110390768

[61] Van de Mortel TF. Faking it: social desirability response bias in self- report research. Aust J Adv Nurs. (2008) 25:40–8.

[62] Bombak AE. Self-rated health and public health: a critical perspective. Frontiers. Public Health. (2013) 1:15. doi: 10.3389/fpubh.2013.00015 PMC3855002 24350184

[63] Onawola RST LaVeist TA. Subjective health status as a determinant of mortality amongst African-American elders. J Natl Med Assoc. (1998) 90:754–8. PMID: 9884495 PMC2608424

[64] Idler EL Benyamini Y. Self-rated health and mortality: a review of twenty-seven community studies. J Health Soc Behav. (1997) 38:21–37. doi: 10.2307/2955359, PMID: 9097506

